# Randomised clinical study: inulin short‐chain fatty acid esters for targeted delivery of short‐chain fatty acids to the human colon

**DOI:** 10.1111/apt.13749

**Published:** 2016-07-28

**Authors:** T. Polyviou, K. MacDougall, E. S. Chambers, A. Viardot, A. Psichas, S. Jawaid, H. C. Harris, C. A. Edwards, L. Simpson, K. G. Murphy, S. E. K. Zac‐Varghese, J. E. Blundell, W. S. Dhillo, S. R. Bloom, G. S. Frost, T. Preston, M. C. Tedford, D. J. Morrison

**Affiliations:** ^1^ Stable Isotope Biochemistry LaboratoryScottish Universities Environmental Research Centre (SUERC)East KilbrideUK; ^2^ School of ScienceUniversity of the West of ScotlandHamiltonUK; ^3^ Nutrition and Dietetic Research GroupHammersmith CampusFaculty of MedicineImperial College LondonLondonUK; ^4^ School of MedicineCollege of Medical Veterinary and Life ScienceUniversity of GlasgowGlasgowUK; ^5^ Section of Endocrinology and Investigative MedicineHammersmith CampusFaculty of MedicineImperial College LondonLondonUK; ^6^ Institute of Psychological SciencesUniversity of LeedsLeedsUK

## Abstract

**Background:**

Short‐chain fatty acids (SCFA) produced through fermentation of nondigestible carbohydrates by the gut microbiota are associated with positive metabolic effects. However, well‐controlled trials are limited in humans.

**Aims:**

To develop a methodology to deliver SCFA directly to the colon, and to optimise colonic propionate delivery in humans, to determine its role in appetite regulation and food intake.

**Methods:**

Inulin SCFA esters were developed and tested as site‐specific delivery vehicles for SCFA to the proximal colon. Inulin propionate esters containing 0–61 wt% (IPE‐0–IPE‐61) propionate were assessed *in vitro* using batch faecal fermentations. In a randomised, controlled, crossover study, with inulin as control, *ad libitum* food intake (kcal) was compared after 7 days on IPE‐27 or IPE‐54 (10 g/day all treatments). Propionate release was determined using ^13^C‐labelled IPE variants.

**Results:**

*In vitro*, IPE‐27–IPE‐54 wt% propionate resulted in a sevenfold increase in propionate production compared with inulin (*P* < 0.05). *In vivo*, IPE‐27 led to greater ^13^C recovery in breath CO_2_ than IPE‐54 (64.9 vs. 24.9%, *P* = 0.001). IPE‐27 also led to a reduction in energy intake during the *ad libitum* test meal compared with both inulin (439.5 vs. 703.9 kcal, *P =* 0.025) and IPE‐54 (439.5 vs. 659.3 kcal, *P* = 0.025), whereas IPE‐54 was not significantly different from inulin control.

**Conclusions:**

IPE‐27 significantly reduced food intake suggesting colonic propionate plays a role in appetite regulation. Inulin short‐chain fatty acid esters provide a novel tool for probing the diet–gut microbiome–host metabolism axis in humans.

## Introduction

One major function of the gut microbiota is the metabolism of nondigestible carbohydrates (NDC), which leads to the production of short‐chain fatty acids (SCFA) as the major end‐products. SCFA have been shown to have a range of beneficial effects, including improved immune function, adiposity and glucose regulation albeit the data come almost exclusively from animal models.^1^, ^2^, ^3^, ^4^, ^5^ Comparable mechanistic data in humans are largely lacking because of the difficulty in selectively and controllably manipulating SCFA production in human intervention studies. The discovery of free fatty acid receptors (FFAR 2/3), for which SCFA appear to be the natural ligands, has provided a putative mechanism of action for SCFA and may in part explain some of the beneficial effects observed from dietary NDC consumption.^6^, ^7^, ^8^ High intake of fermentable NDC has been shown to influence body composition in animals,^9^ promote weight loss in humans^10^ and improve glucose homoeostasis in both animals^11^ and man.^12^ NDC ingestion stimulates the release of glucagon‐like peptide (GLP‐1) and peptide YY (PYY), resulting in a reduction in food intake in animals^13^ and humans,^14^ albeit at doses that exceed typical daily NDC intake. SCFA have also been shown to increase GLP‐1 and PYY secretion in animals^15^, ^16^ and in man,^17^ suggesting that SCFA may, at least in part, mediate the effects of NDC on appetite regulation. However, UK NDC intake (major component of dietary fibre), has like many Western societies remained well below the recommended levels, at around 15 g/day (measured as nonstarch polysaccharide in the UK) and 16 g/day dietary fibre in the USA.^18^, ^19^ Alternative ways of optimising colonic SCFA production may be needed.

Of the SCFA produced in the colon, propionate has highest affinity for FFAR 2, and a lower binding affinity for FFAR 3.^7^ Oral propionate supplementation has been investigated in experimental studies of appetite regulation in humans,^20^, ^21^ but its poor organoleptic properties, short‐circulating half‐life and the fact that it is absorbed in the proximal small intestine limit its use as a food supplement targeting large intestinal FFAR 2. To induce large changes in propionate production, while avoiding the unwanted gastrointestinal side effects of high NDC diets, requires a more targeted approach. Recently, we described the first‐in‐man studies showing that propionate, when delivered to the colon, can induce appetite regulation, improve metabolic control and play a role in weight management.^22^ This study was, to the best of our knowledge, the first to translate direct observations from propionate supplementation in animal studies into a randomised, controlled trial in humans, whereby propionate was targeted to the main site of its production, the proximal colon.

Targeting small molecule delivery to the large intestine is challenging because the vector has to avoid absorption in the small intestine, resist digestion by brush‐border and exocrine enzymes and thereafter selectively release its small molecule payload in the large intestine. Techniques for small molecule colonic delivery such as encapsulation, using fermentable polymer coatings,^23^ pH and/or time‐dependant release^24^ and direct instillation, using SCFA enema^25^ are limited to relatively low dosing regimens or are unsuitable for population‐level interventions. The binding affinity between FFAR and SCFA appears adapted for the relatively high SCFA concentrations found in colonic environment, resulting to a key specification of any colonic delivery system for SCFA: it must be capable of inducing significant change in the large colonic SCFA pool. Using observations from sudden death victims, SCFA contents of the caecum were 69.1, 25.3 and 26.1 mmol/kg contents for acetate, propionate and butyrate respectively.^26^ This suggests the caecum alone has a propionate pool size of 1.87 g/kg contents. Combining stoichiometric equations for NDC fermentation^27^ and National Diet Nutrition Survey data^18^ of habitual fibre intake suggests that the average adult 15 g/day nonstarch polysaccharide intake in the UK yields a minimum of 0.4 g of propionate production per day. We therefore set a threshold of delivering >2 g/day propionate to induce a fivefold increase in daily propionate production and broadly equivalent to fermentation of 90 g of NSP per day. SCFA functionalised starches have been used in enteric coating for colon‐specific drug delivery^28^ and the concept was extended to starch SCFA esters as a prodrug delivery system for SCFA to the colon.^29^ However, the choice of starch as a starting material leads to complexities in synthesis, characterisation and determination of SCFA delivery efficacy because of the variable effects of the chemical modifications on the nature and digestibility of the starch itself. Starch solubilisation and functionalisation also requires highly polar organic solvents leading to high purity requirements in downstream processing for a food‐grade material. In the present study, we describe the production and optimisation of inulin propionate esters with favourable organoleptic properties and designed to release propionate within the large intestine to facilitate the study of appetite regulation. Our choice of inulin was predicated on the fact that inulin is water soluble and amenable to aqueous‐based functionalisation and that its gastrointestinal handling had been well characterised.^30^, ^31^ Our aim was therefore to develop suitable inulin propionate esters (IPE) for highly efficient colonic propionate delivery and to assess the effects of IPE variants on appetite and food intake.

## Materials and methods

Inulin was used as the carrier (Beneo HP; DSKH, London, UK). Inorganic reagents (Sigma‐Aldrich, Dorset, UK) and propionic anhydride (Acros Chemicals/Fisher, Loughborough, UK) were of the highest purity available. Activated charcoal [4 mm pellets from coconut shell (Eurocarb, Bristol, UK)] was used as the primary clean‐up column. Dialysis was carried out overnight using a 1000 MWCO tubular membrane, which could be sealed at both ends (Spectra‐Por, Breda, The Netherlands). All water used in synthesis, column conditioning and washing was deionised with >15 MΩ resistivity.^13^C‐labelled (1‐^13^C)_2_ propionic anhydride was purchased from Cambridge Isotopes Limited (CK Gas, Hampshire, UK) as a custom synthesised product.

### Synthesis and characterisation of IPE

The following describes the route to IPE with a target degree of esterification of 0.8, on average every 1 in 1.25 fructose moieties will have one hydroxyl group esterified. This equates to a propionate loading of 27 wt%. The degree of esterification (propionate loading) can be varied by the amount of propionic anhydride added and the following is provided by way of exemplar of a route to food‐grade IPE. Inulin HP (450 g; Beneo‐Orafti Food Ingredients, Tienen, Belgium) was dissolved in 2 L deionised water and transferred to a 3 L water‐cooled jacketed reactor with overhead stirring and continuous pH monitoring and allowed to cool to ~ 25 °C. Propionic anhydride (360 mL, 2.8 moles) and NaOH (400 mL, 25% w/v) were set up in dropper funnels above the reactor. The pH was adjusted to 8.25 by dropwise addition of NaOH and the addition of propionic anhydride commenced when the temperature in the reaction mixture was <20 °C. The rate of addition of reagents was such that the pH remained between 8 and 8.5 and the temperature remained <20 °C. Once addition of propionic anhydride was complete the reaction mixture was allowed to pH stabilise and thereafter adjusted to pH 2 with concentrated HCl. Immediately, the reaction mixture was allowed to flow through an activated carbon (4 mm granular coconut shell; Eurocarb) column which had been thoroughly washed and conditioned with 0.2 M HCl. The column contained ~ 1 kg activated charcoal and the reaction mixture was allowed to flow through at approximately 0.5 L/h. Activated carbon has a higher affinity and sequestration capacity for propionic acid compared with the anionic form–propionate.^32^ The reaction mixture recovered from the column was adjusted to pH 2 with concentrated HCl and passed through a second column prepared in an identical fashion to the first column. The reaction mixture was subjected to overnight dialysis in dialysis tubing (Spectra/Por 6, 1000 MWCO; Spectrum Europe B.V., Breda, the Netherlands). The reaction mixture was collected and again adjusted to pH 2 before spray drying (Buchi, Oldham, UK) in an inert N_2_ gas flow. The flow of liquid, N_2_ and nebuliser temperature were such that an outlet temperature of ~100 °C was maintained.

IPE was characterised by infrared spectroscopy for the presence of an ester bond, GC‐FID to establish levels of free propionate, salt content and heavy metal content, and subjected to microbiological testing of the final product (details available in supporting material).

### Microbiota metabolism of IPE *in vitro*

The fermentation profiles of inulin (control) and IPE variants ranging from 10% to 61% loading (equating to a degree of esterification range of 0.25–2.5) were tested using batch faecal cultures.^33^ Inulin was selected as control to account for SCFA production from the NDC backbone. Faecal samples were collected from three healthy volunteers (who had no history of gastrointestinal complaints and were antibiotic free for 6 months prior to faecal collection) and prepared separately in triplicate for each substrate. The composition of the faecal batch fermentation systems is described in detail in supporting information. The absolute production of SCFA (at 24 h), molar ratios and yield of propionate from IPE (calculated after subtracting propionate production from inulin) were calculated. The efficiency (% of theoretical propionate yield) was also calculated.

The ability of a number of esterase enzymes to release propionate was tested using the standard esterase kit available from Sigma‐Aldrich (Poole, UK). Briefly, IPE, ethyl propionate (positive control) and water (negative control) were incubated in phosphate buffer along with esterases from *Candida lipolytica*,* Mucor miehei*,* Pseudomonas fluorescens* (recombinant from *E. coli*), *Streptomyces diastochromogenes* (recombinant from *E. coli*), horse liver and hog liver. Propionate release was determined by GC. Detailed methods are available in supporting material.

### IPE palatability

A short consumer palatability study was undertaken to assess the organoleptic properties of IPE incorporated into food products. This feasibility study aimed to determine if IPE could be practically incorporated into food products masking the poor organoleptic properties of free propionate. Full details on the methodology are available in supporting information.

## Randomised, crossover study to determine effects of IPE variants on food intake

### Subjects

Overweight healthy males were the target cohort for this study to mitigate against the effects of the menstrual cycle on appetite regulation and food intake. Nine healthy males were recruited to characterise which IPE preparation optimises delivery to and release of propionate in the colon. The characteristics of the cohort were (mean ± S.E.M.) age (38 ± 9 years), weight (98.36 ±3.0 kg) and body mass index (BMI; 29.8 ± 1.5 kg/m^2^) respectively.

Participants were recruited via local advertising. The inclusion criteria for all three investigations were age 21–65 years and BMI range 25–35 kg/m^2^. Exclusion criteria were smoking, substance abuse, use of medications, a change in body weight >5 kg in the previous 3 months, medical or psychiatric illness, assessed by a self‐reported medical questionnaire. The study was approved by College of Medical, Veterinary and Life Sciences ethics committee for nonclinical research in human volunteers at the University of Glasgow. All studies were carried out in accordance with the Declaration of Helsinki. The study was registered with ClinicalTrials.gov (NCT02229500).

### Study design

This study was a four‐wing randomised crossover design where each volunteer acted as their own control. The study aimed to characterise an IPE preparation that maximises the bioavailability of propionate to the colon. After a familiarisation/screening visit which also served as a baseline study day, volunteers were randomly allocated to one of three treatments using a random number generator. The principal grant holder who did not carry out the experimental trials performed the random allocation. The allocation of codes for each supplement and their respective participant ID was sent to the investigator carrying out the experimental trials, who then enrolled participants with the allocated supplement. Both the investigator running the experimental trials and the participants were blinded to the contents of the supplement. The location for the experimental visit was the Clinical Research Facility (CRF) at Glasgow Royal Infirmary. Participants visited the CRF on four occasions throughout the study. The screening trial acted as a familiarisation trial and after consuming a supplement (detailed later) for 1 week, participants re‐visited the CRF on day 7 of supplementation. At least 2‐week washout period was allowed between supplements. Supplementation periods took place under free‐living conditions and washout involved normal habitual diet and lifestyle. Participants were asked to record their food intake for 3 days prior to the CRF visit and replicate this prior to each study visit. As a measure of compliance for supplement intake, subjects were requested to return empty sachets to the investigator. The primary outcome measure for this study was area under the curve of breath ^13^CO_2_ enrichment (a proxy for the extent of propionate release). Participants were also asked to provide a stool sample during or immediately after each visit to the CRF to assess stool ^13^C output. Urine was collected for 24 h post‐dose to assay urinary ^13^C output.

At the end of the screening/familiarisation trial, participants were provided with the coded sachets containing the supplement and were asked to consume this with breakfast for 6 days. The supplement was either IPE‐27, IPE‐54 or inulin, each containing 10 g/day single dose. On day 7, participants attended the CRF for their next visit, which lasted 8 hours. At the end of the 2nd visit, participants were provided with the next supplement in identical sachets and were advised to undergo a minimum 14‐day wash out period, prior to consumption of the supplement (consumption started on day 15). Thus after another 7 days (on day 21), volunteers reported to the CRF for a repeat measurement day. Following a further 14‐day washout, participants consumed the final supplement and returned to the CRF 7 days after supplementation for repeat measurement day. Washout often lasted longer than 14 days to accommodate volunteer lifestyle. On the CRF study days consuming IPE variants, a sachet containing the respective unlabelled IPE mixed with a ^13^C‐labelled IPE (300 mg of ^13^C IPE‐27 or 100 mg of ^13^C IPE‐54 providing similar amounts of tracer) of identical composition was provided with a standard breakfast. Blood was collected via indwelling venous cannula (inserted into an antecubital vein) and baseline blood samples were collected into heparin‐coated tubes containing 0.2 mL of aprotinin (Sigma‐Aldrich) at −10 min and 0 min and then at 60 min intervals up to 7 h assess plasma PYY and GLP‐1 concentrations (further details available in supporting material).

### *Ad libitum* food intake and appetite

On each study day, participants were offered standard breakfast, a balanced meal for lunch at 4 h after breakfast and at the end of the study day (at 8 h following breakfast), food intake was assessed by offering a free buffet meal. Breakfast included a plain scone, jam, butter and orange juice (calories, 266 kcal; protein, 2.8 g; fat 6.5 g, CHO, 48.9 g), while lunch included a cheese sandwich, an orange, orange juice and strawberry yoghurt (calories, 676.9 kcal; protein, 28.6 g; fat, 23 g; CHO, 92,7 g). The ad libitum meal included a selection of sandwiches prepared by the catering service of the hospital, toffee yoghurt, an orange and an orange juice (calories, 1440 kcal; protein, 52 g; fat, 46.5 g, CHO, 209 g). The meals were weighed before and after consumption with the use of a digital kitchen scale. The nutritional information for all foods was offered by the catering service and thus accurate energy and macronutrient intake, based on the final weight consumed, was calculated for all meals. Furthermore, participants were asked to replicate their evening meal on the evening preceding the trial day. At baseline and every 30 min, participants were asked again to rate their feelings of hunger and fullness using visual analogue scales (VAS; further details available in supporting material).

### ^13^C analyses

CO_2_ was collected serially over 12 h followed by one further sample collection at 24 h (to verify return to natural abundance levels) by exhaling alveolar breath through a straw into Exetainers (Labco, Ceredigion, UK). ^13^CO_2_ enrichment was determined by isotope ratio mass spectrometry (IRMS). ^13^CO_2_ above baseline (post‐dose – pre‐dose ^13^CO_2_; ppm xs ^13^C) and cumulative ^13^CO_2_ excretion were also calculated. CO_2_ production rate was estimated from body surface area and a physical activity level factor of 1.3 was used to allow calculation of cumulative ^13^C dose recovered (%).^34^ Stool and urine ^13^C output were also determined using IRMS (detailed methods in supporting material).

### Data Analysis

Results are expressed as mean ± s.d. or S.E.M. (where indicated). Results were compared by anova with *post hoc* analysis. Statistical analysis was conducted on spss 18 (Chicago, IL, USA).

## Results

### Synthesis and characterisation of IPE

The yield of IPE from inulin was ~70%. The major losses of product occurred on the column, when precipitation started to occur, and in the spray dryer, where product collected on the vaporisation chamber and filter. Post‐reaction pH control was necessary to maintain propionate in the acidic form to facilitate its removal on the column. In addition, the spray drying conditions promoted volatilisation of free propionic acid, further purifying the product. The degree of esterification of the final product was 0.74 ± 0.02, with a free propionate content (of all available propionate) of 1.25 ± 0.30% (see supporting material for further information on IPE purity).

### *In vitro* and *in vivo* results

**Figure 1:**
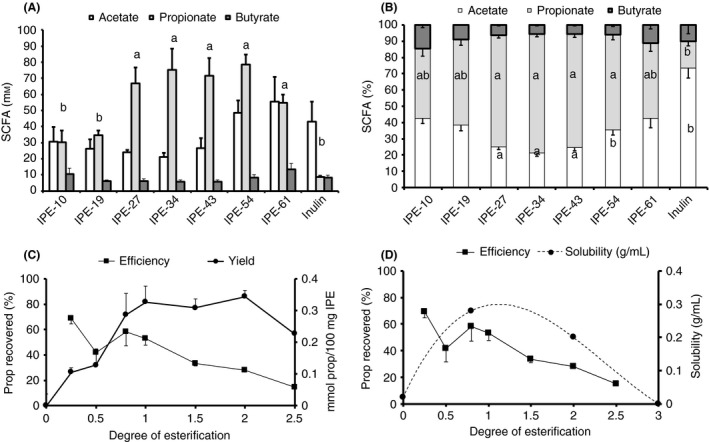
Mean (S.E.M.) absolute SCFA production (A), molar ratios (B), efficiency and yield of propionate (C) and efficiency and solubility (D) from IPE with propionate content ranging from 0 to 61 wt% in faecal fermentations (*n* = 3). Treatments were compared, for each SCFA, using anova and significant differences from inulin control (a, *P* < 0.05) and IPE d_e_ = 1.0 (b, *P* < 0.05) are indicated. A polynomial fit was used to model the solubility vs. degree of esterification data with the following equation: *y* = 0.045*x* ^3^−0.32*x* ^2^ + 0.554*x* + 0.02, *R* ^2^ = 1.

Not all IPE preparations were equally well fermented. Propionate production in faecal fermentations was significantly higher in variants containing 27–54 wt % propionate (IPE‐27–IPE‐54; Figure 1A,B). IPE variants in the range 27–54% propionate yielded similar levels of propionate and IPE‐27 appears to be the most efficient at releasing propionate (variant with maximal efficiency and yield; Figure 1C) and this decrease in efficiency appeared to mirror the decrease in solubility with increasing degree of esterification (Figure 1D). Propionate release from IPE incubated with esterases was low indicating that, at least for the species tested, de‐esterification appeared limited (Figure S1 in Data S1).

**Figure 2:**
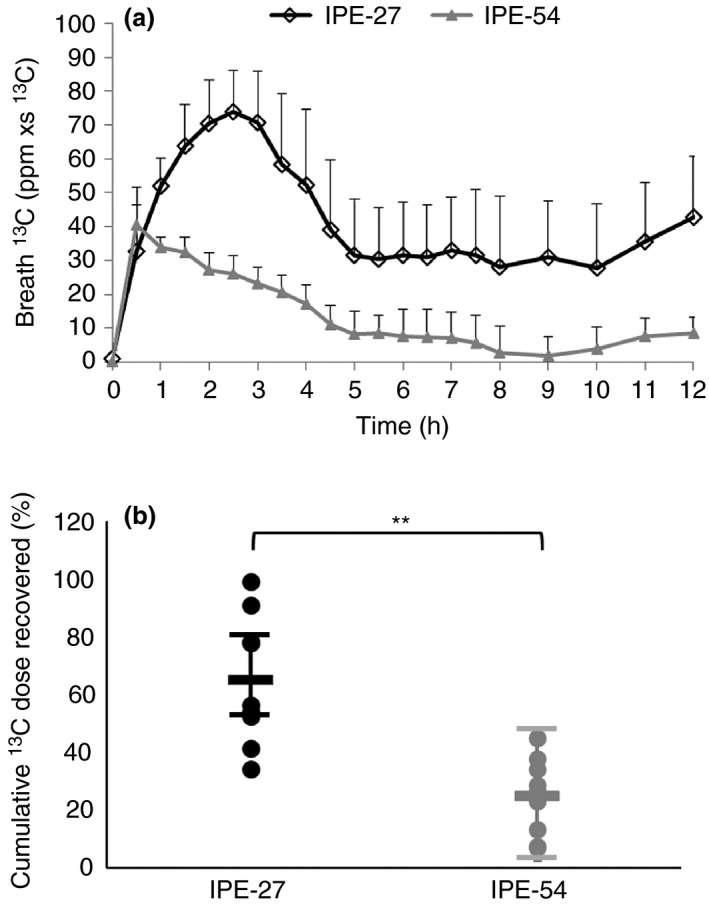
Profile of ^13^ CO_2_ recovery (a) and mean (s.d.) cumulative ^13^C recovery (b) from propionate release and oxidation following supplementation with the IPE‐27 and IPE‐54. Breath ^13^ CO_2_ excretion for IPE‐27 was higher (from 1 h onwards) compared with IPE‐54 (*P <* 0.05). IPE‐27 led to significantly greater ^13^C recovery in breath CO_2_ (64.9 vs. 24.9%, *P =* 0.001) compared with IPE‐54.

The data obtained from human experiments indicated that recovery of ^13^C in breath continued for more than 12 h (Figure 2a), but appeared to have returned almost to baseline abundance by 24 hrs (data not shown). IPE‐27 led to significantly greater ^13^C recovery in breath CO_2_ (64.9 vs. 24.9%, *P* = 0.001; Figure 2b) compared with IPE‐54. Only fasting PYY was elevated when comparing treatments with control, with a significantly lower incremental area under the curve (iAUC) observed for IPE‐54 compared with inulin control for PYY only (Results section and Figures S2a–d in Data S1). There was no difference in stool ^13^C recovery (Figure S2 in Data S1) which equated to a mean (SEM) recovery of 0.13 (0.11) and 0.39 (0.16) % of administered tracer for IPE‐54 and IPE‐27 respectively using average daily stool output of 106 g/day for UK adults.^35^ Similarly, there was no difference in urine ^13^C enrichment, which barely deviated from isotopic natural abundance (Figure S3 in Data S1).

**Figure 3:**
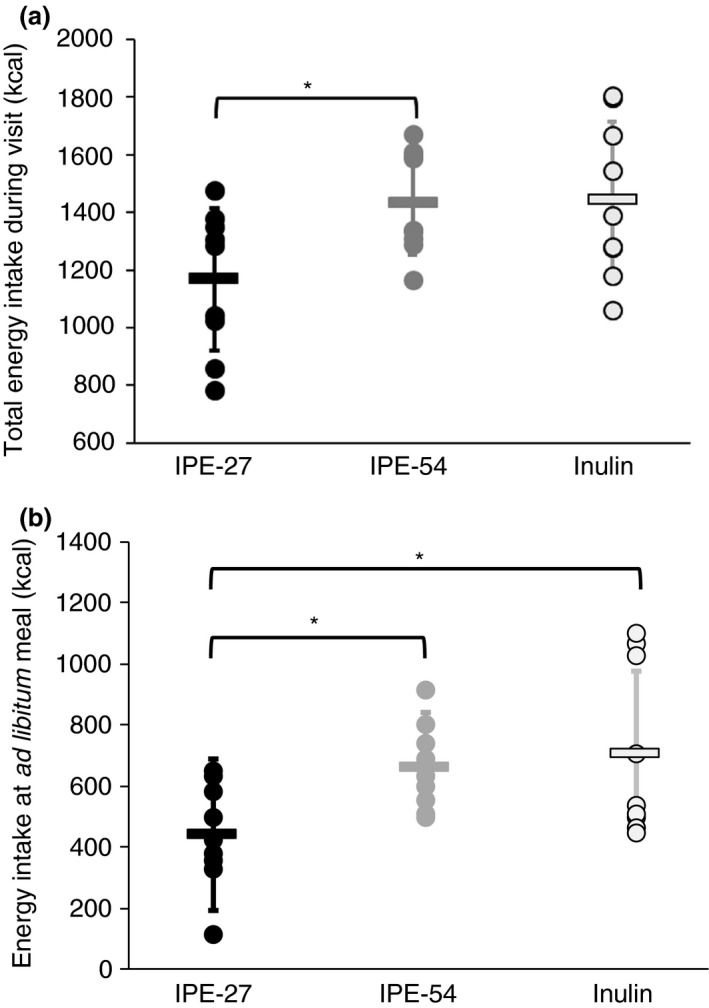
Mean (s.d.) total energy intake (a; kcal) and *ad libitum* buffet meal energy intake (b; kcal) during the experimental day following 7‐day supplementation with IPE‐27, IPE‐54 or inulin. IPE‐27 led to significantly lower total energy intake compared with IPE‐54 (1167.6 vs. 1432.9 kcal, *P = 0.016) but only a trend towards lower intake compared with inulin control (1167.6 vs. 1444.6 kcal, *P =* 0.076). IPE‐27 led to a significant reduction in energy intake during the *ad libitum* test meal compared with both inulin (439.5 vs. 703.9 kcal, *P =* 0.025) and IPE‐54 (439.5 vs 659.3 kcal, *P =* 0.025). IPE‐54 was not significantly different from inulin control.

IPE appears palatable to participants and indistinguishable from inulin when consumed in certain foods (Figure S4 in Data S1). This would appear to discount an adverse organoleptic effect from consuming IPE. IPE‐27 led to a significant reduction in energy intake during the *ad libitum* test meal compared with both inulin (439.5 vs. 703.9 kcal, *P =* 0.025) and IPE‐54 (439.5 vs. 659.3 kcal, *P =* 0.025; Figure 3a). IPE‐54 was not significantly different from inulin control for the *ad libitum* test meal. IPE‐27 led to significantly lower total energy intake compared with IPE‐54 (1167.6 vs. 1432.9 kcal, *P = 0.016) but only a trend towards lower intake compared with inulin control (1167.6 vs. 1444.6 kcal, *P =* 0.076; Figure 3b). There were no associated differences observed in the visual analogue scales (Figures S5 and S6 in Data S1).

## Discussion

We have designed a process to produce kg quantities of an inulin SCFA ester with desirable characteristics for targeted colonic delivery of SCFA to the colon. For inulin propionate ester (IPE), our study demonstrates a nonlinear relationship between propionate release *in vitro* and the degree of IPE esterification. Our study further demonstrated that an IPE variant with 27% (wt/wt) propionate loading is more efficient at releasing propionate when compared with an IPE variant with 54% loading and that the decrease in efficiency of IPE variants mirrors the decrease in solubility of IPE as more hydroxyl groups are replaced by propionyl groups. The fermentation profile of IPE‐27 confirmed our earlier findings^22^ and the present study demonstrates that higher degrees of esterification (54%) does not appear to lead to increased propionate release in the colon, mirroring our *in vitro* data. Finally, consumption of IPE‐27 was associated with appetite suppression as measured through *ad libitum* food intake, whereas IPE‐54 did not suppress food intake.

We have selected to work with IPE‐27 because it is the IPE variant with the highest degree of esterification that remains water soluble all the way through the downstream processing. In previous work, we postulated that 10 g IPE‐27 releases an amount of propionate which approximates to a 2.5‐fold increase in daily colonic propionate production (based upon stoichiometric propionate production from habitual dietary fibre intake).^22^ These are levels sometimes consumed in populations with ‘high fibre’ intake with no detrimental effects. Moreover, in a 6‐month intervention with IPE‐27 (10 g/day), participants reported limited side effects and similar to the inulin control intervention.^22^

A previous study based on starch esters has suggested that a targeted approach is potentially feasible for modulating colonic SCFA.^29^ Direct evidence of regional SCFA availability is, however, difficult to monitor without an isotopic approach. Starch is also a complex carbohydrate, and different starch fractions have different physiological fates. Some fractions are digestible and some resistant, becoming available for fermentation.^36^ Esterification of starch by chemical means is nonspecific, resulting in random esterification of all starch fractions. Clarke *et al*. estimated that 30% of bound butyrate from their ester preparation was released in the small intestine and ~60% released in the colon. SCFA have complex physiological roles, involving receptor‐mediated responses in the intestine and metabolic effects in other extra‐intestinal organs, which may be receptor independent.^37^, ^38^, ^39^ Delineating the mechanism of action in human studies therefore requires discrete site‐directed delivery. We therefore set a high threshold for delivery of propionate specifically to the colon. We have concentrated on using an inulin carrier molecule because it has a simple linear structure, desirable chemical characteristics (including water solubility) and the extensive published data regarding its low level of digestion in the human gastrointestinal tract.^40^, ^41^ We have previously reported that propionate derived from IPE‐27 appears in the plasma propionate pool.^22^

There is considerable interest in the modulation of specific SCFA in the colon because it is now recognised that they may have roles beyond simply acting as metabolic substrates. The role of SCFA in metabolic health, appetite regulation and energy homoeostasis has recently been extensively reviewed.^42^, ^43^, ^44^, ^45^ FFAR 2 and 3 are expressed in the terminal ileum and colon, and are activated by SCFA at the concentration range found in the colon, suggesting SCFA may mediate their effects via these receptors. SCFA‐driven anorexigenic gut hormone production has been demonstrated both in animals^15^, ^16^ and in man.^17^ Previous work has shown that high‐fibre diets associated with SCFA production induce improved glucose tolerance at subsequent meals; the so‐called ‘second meal effect’.^46^, ^47^, ^48^ Our data also demonstrate that increased propionate release in the colon leads to a reduction in energy intake during an *ad libitum* buffet meal. We have shown previously a similar finding which in an acute setting appears to be mediated by the anorexigenic gut hormones GLP‐1 and PYY.^22^ In the present study, however, only fasting PYY was significantly elevated (for both IPE treatments) but not postprandial hormone release compared with control (although a lower iAUC was observed for IPE‐54 compared with control). The breath ^13^CO_2_ excretion data suggests an extended timescale for propionate release from IPE with an apparent increase at 10–12 h post‐dose. This elongated release of propionate may be one explanation for the elevated fasting response but further work is necessary to determine the dynamics of SCFA production and gut hormone response as we have previously observed a diminishing gut hormone response over time on IPE supplementation.^22^ An interesting finding arising from the present study is that IPE variants with an intermediate degree of esterification led to a lower total energy intake and *ad libitum* energy intake compared with a higher degree esterification variant. This coincides with the finding that more propionate became available from IPE‐27, as demonstrated by the greater ^13^CO_2_ recovery in the breath compared to IPE‐54. Our previous work demonstrated that 82.9 ± 2.3% of the ^13^C recovered in breath over 24 h appeared coincident with or after breath H_2_ onset release when IPE‐27 was consumed.^22^ The early peak in breath ^13^CO_2_ observed with IPE‐54 was likely due to the higher free (labelled) propionate content (details in supporting material) compared with IPE‐27. Free propionate is rapidly oxidised to breath ^13^CO_2_ when given orally (data not shown) in an analogous fashion to ^13^C acetate which is used in gastric emptying breath tests.^49^ Interestingly, this divergence in tracer recovery in breath was not mirrored in stool output. There are several potential limitations to the stool collection. Firstly, only a single post‐tracer stool sample was collected. The particularly onerous burden on participants of 3‐day or 5‐day stool collection was a high barrier to participation in the study and unrealistic in this setting. Thus, a significant fraction of the tracer may have been excreted in the later stools particularly if propionate effects gut transit times. There is however conflicting evidence to support the role of SCFA as modulators of colonic activity and thus whether propionate release influenced gut transit is unknown.^15^, ^50^ We have previously observed little effect of IPE on gastric emptying.^22^ IPE‐54 is more lipophilic than IPE‐27 and therefore uptake and sequestration into micelles and selective lymphatic uptake is a potential mechanism leading to reduced bioavailability IPE to colonic fermentation. IPE (average MW > 5000 Da) is unlikely to be absorbed directly either by active or passive absorption mechanisms across the gut wall. This specific inulin product was chosen because of the absence of mono‐ and disaccharides. Nanoparticles can be sequestrated in the gut by selective lymphatic uptake and is dependent on their lipophilicity.^51^ Urine ^13^C analysis suggests that little tracer was excreted in the urine because the isotopic abundance in urinary carbon barely deviated from natural abundance.

Taken together this study demonstrates that the composition of inulin SCFA esters is critical to their intended use. Increasing the propionate content of the ester, on the face of it desirable to increase the delivery of propionate to the colon, is not linearly related to propionate delivery to the large intestine. The properties of the molecule appear to be critical to manipulating SCFA production in the colon and each inulin SCFA ester should be tested using isotopic profiling to ensure appropriate release profile. Water solubility appears to be an important factor in determining the release kinetics of SCFA from the ester. This study further observed significant reductions in energy intake at a buffet meal when consuming IPE‐27, but not IPE‐54, compared with inulin control, by investigating the effects of different propionate content in IPE intake on appetite, reflected by energy intake consumed in an *ad libitum* meal. The selective manipulation of propionate and comparison with inulin as control provides strong evidence for propionate as a regulatory signal from the colon involved in appetite regulation^22^ and suggests that IPE‐27 is an efficient inulin propionate ester to induce regulation of appetite and food intake. This study demonstrates that selective modulation of individual SCFA can be achieved by inulin SCFA esters and that SCFA may have important regulatory roles in human physiology. Further work is needed to fully elucidate the roles of other SCFAs in human health.

## Authorship

*Guarantor of the article*: Douglas J. Morrison

*Author contributions*: Polyviou, Chambers, Viardot and Psichas conducted the human intervention studies. Harris, Simpson, Edwards and Morrison designed and conducted the *in vitro* studies. Macdougall, Jawaid, Tedford, Preston, Frost and Morrison developed and produced the inulin propionate ester. Murphy, Zac‐Varghese, Blundell, Dhillo, Bloom, Frost, Preston, Tedford and Morrison designed the study and secured funding. Polyviou, Chambers, Frost and Morrison produced the initial draft of the paper and Chambers, Frost and Morrison produced the revised version of the paper.

All authors approved the final version of the manuscript.

## Supporting information


**Figure S1**. Propionate production from incubation of IPE‐27 and ethyl propionate (EP; positive control) from esterases from *Candida lipolytica*,* Mucor miehei*,* Pseudomonas fluorescens* (recombinant from *E. coli*), *Streptomyces diastochromogenes* (recombinant from *E.coli*), horse liver and hog liver. Data are expressed and % of theoretical propionate recovery.
**Figure S2.** Plasma PYY concentration versus time (Figure 2), plasma PYY AUC, AUC_0–180_, AUC_180–420_ and iAUC (Figure 2B) and plasma GLP‐1 concentration versus time (Figure 2C), plasma GLP‐1 AUC, AUC_0–180_, AUC_180–420_ and iAUC (Figure 2D). * indicates significant differences (*P* < 0.05) versus inulin control treatment.
**Figure S3.** Fractional stool ^13^C recovery x 100 (%) per gram wet weight. Participants followed a 6‐day supplementation regimen followed with replacement of the dose on day 7 with either 100 mg of ^13^C‐IPE‐54 or 300 mg of ^13^C‐IPE‐27. In both studies, the carboxyl carbon (1‐^13^C) of the propionyl moiety was labelled. Data are Mean ± SEM; *n* = 9.
**Figure S4**. 24 hr urine ^13^C enrichment (abundance) after ingestion of ^13^C labelled IPE‐27 and IPE‐54 from the cross over trial. Data are expressed as mean (SEM) as d^13^C (^0^/_00_).
**Figure S5.** Comparison of the organoleptic properties of control, inulin and inulin propionate (IPE‐27) in chocolate milkshake (**A**) and heated tomato soup (**B**) (*n* = 15). Values are mean and SEM scores for visual analog scale assessment of how pleasant the material was to consume. **P < 0.01 and *P < 0.05.
**Figure S6.** Subjective ratings of appetite following 7 day supplementation with inulin, IPE‐54 and IPE‐27. Ten grams of inulin, IPE‐54 or IPE‐27 was ingested with an identical evening meal and with a standardized breakfast the following morning. **A.** *Hunger*,** B.** *Satiety*,** C.** *Fullness*, and **D.** *Prospective food consumption (PFC),* **E**. *Desire to eat*. Ratings were made using 100 mm visual analogue scales (VAS), with extreme statements anchored at each end of the rating scale (e.g. 0 mm *Not at all hungry*, 100 mm *Extremely hungry*). Data are mean ± SEM; *n* = 9.
**Figure S7.** Visual analogue scale area under the curve for the period 0–480 min (AUC_0–480_; mm × min) data for each rating is also shown. Data are mean ± SEM; *n* = 9.
