# Supplementary material for: Randomised clinical study: inulin short‐chain fatty acid esters for targeted delivery of short‐chain fatty acids to the human colon
Source: Aliment Pharmacol Ther. 2016 Jul 28;44(7):662–72. doi: 10.1111/apt.13749 (PMC5026196; doi:10.1111/apt.13749)
Supplement: Supplementary file 1 — Figure S1. Propionate production from incubation of IPE‐27 and ethyl propionate (EP; positive control) from esterases from Candida lipolytica, Mucor miehei, Pseudomonas fluorescens (recombinant from E. coli), Streptomyces diastochromogenes (recombinant from E.coli), horse liver and hog liver. Data are expressed and % of theoretical propionate recovery. Figure S2. Plasma PYY concentration versus time (Figure 2), plasma PYY AUC, AUC0–180, AUC180–420 and iAUC (Figure 2B) and plasma GLP‐1 concentration versus time (Figure 2C), plasma GLP‐1 AUC, AUC0–180, AUC180–420 and iAUC (Figure 2D). * indicates significant differences (P < 0.05) versus inulin control treatment. Figure S3. Fractional stool 13C recovery x 100 (%) per gram wet weight. Participants followed a 6‐day supplementation regimen followed with replacement of the dose on day 7 with either 100 mg of 13C‐IPE‐54 or 300 mg of 13C‐IPE‐27. In both studies, the carboxyl carbon (1‐13C) of the propionyl moiety was labelled. Data are Mean ± SEM; n = 9. Figure S4. 24 hr urine 13C enrichment (abundance) after ingestion of 13C labelled IPE‐27 and IPE‐54 from the cross over trial. Data are expressed as mean (SEM) as d13C (0/00). Figure S5. Comparison of the organoleptic properties of control, inulin and inulin propionate (IPE‐27) in chocolate milkshake (A) and heated tomato soup (B) (n = 15). Values are mean and SEM scores for visual analog scale assessment of how pleasant the material was to consume. **P < 0.01 and *P < 0.05. Figure S6. Subjective ratings of appetite following 7 day supplementation with inulin, IPE‐54 and IPE‐27. Ten grams of inulin, IPE‐54 or IPE‐27 was ingested with an identical evening meal and with a standardized breakfast the following morning. A. Hunger, B. Satiety, C. Fullness, and D. Prospective food consumption (PFC), E. Desire to eat. Ratings were made using 100 mm visual analogue scales (VAS), with extreme statements anchored at each end of the rating scale (e.g. 0 mm Not at all hungry, 100 mm Ext [file APT-44-662-s001.docx]

**SUPPLEMENTARY MATERIAL**

**MATERIALS AND METHODS**

**Characterization of Inulin Propionate Ester**

#### *Infrared spectroscopy*

Infrared (FT-IR, Perkin Elmer, Cambridgeshire UK) spectra were recorded using KBr discs. KBr discs were formed by grinding 2-3 crystals of product in an agate pestle and mortar with 100-200 mg KBr. The disc was formed in a hydraulic press.

*Analysis of propionate content*

Inulin Propionate Ester (IPE; 100 mg) was dissolved in 2 ml water containing 10 mM butyric acid as internal standard (IS). Once completely dissolved, 200 μL of the solution was treated with 100 μL of concentrated orthophosphoric acid followed immediately by ether extraction (1 ml), and another 200 μL of the solution was treated with 100 μL of concentrated orthophosphoric acid and heated at 80 °C for 1 hour before being extracted with 1 ml ether. The ether extracts were decanted to clean vials ready for gas chromatography (GC) analysis. Propionate and butyrate were quantified by GC analysis (HP5790, Palo Alto, USA) using a ZB-WAX column (30 m × 0.32 mm × 0.25 μm; Phenomenex, Cheshire UK) and He as carrier gas (1.8 ml/min) in split injection (~50:1 split ratio). The temperature program started at 40 °C and was held for 1 minute before ramping at 10 °C/min to 200 °C. Detection was carried out by flame ionization detection (FID). External standards were run daily to calculate the FID response factor and peak area ratios extracted to an Excel spreadsheet for data processing. Propionate concentration was calculated relative to the IS and the yield of free and total propionate calculated per g ester from the different treatments.

*Salt content*

Salinity of the reaction mixture pre- and post- dialysis was measure using a commercially available salinity meter (Tecpel Pocket Salinity Meter – 850, Digital Meters, UK). The meter was calibrated against gravimetrically prepared standards of NaCl in water.

*Heavy metals screening*

Toxic heavy metals (As, Cr, Cd & Pb) content was measured against an external calibrating standard by inductively coupled plasma-optical emission spectroscopy (ICP-OES; Optima 7300 DV, Perkin Elmer Cambridgeshire UK). Inulin and IPE samples were dissolved in concentrated nitric acid and diluted 1:100 before analysis with reference to the external calibrant. Results (μg/g) were expressed as determined by the instrument.

*Microbiological screening*

Microbiological quality of 12 random IPE batches produced over the period of ~ 1 year was tested externally by an accredited food testing laboratory (Alcontrol Laboratories, Bellshill, UK). The samples were tested for aerobic colony counts (cfu/g), enterobacteriaceae (cfu/g), E. Coli (cfu/g) and Listeria (in 25 g).

*In vitro fermentations*

Fecal fermentation systems consisted of the fermentation medium (2.25g tryptone in 450 ml of distilled water), 112.5 μl of micromineral solution (13.2 g of CaCl_2_, 10.0 g of MnCl_2_, 1.0 g CoCl_2_, FeCl_3_ made up to 100ml with distilled water), 225 ml of macromineral solution (2.85 g Na_2_HPO_4_, 3.1 g KH_2_PO_4_, 0.3 g MgSO_4_ made up to 500 ml with distilled water), 225 ml of buffer solution (2 g NH_4_HCO_3_, 17.5 g NaHCO_3_ made up to 500 ml with distilled water) and 1 ml of 0.1 % (w/v) resazurin solution (a redox indicator). This medium was adjusted to pH 7 using 6M HCl, after which it was sterilized by boiling for 5 minutes. Reducing solution prepared on the day of fermentation (312.5 mg cysteine hydrochloride, 2 ml 1M NaOH, 312.5 mg sodium sulfide, and 47.5 ml distilled water) was added at 0.5 ml per 10 ml of medium after the solution was cooled to 37°C under oxygen-free nitrogen (OFN) until anaerobic conditions were achieved as indicated by a color change from pale indigo to colorless. A 32% fecal slurry was prepared for each subject in 66.6 mM phosphate buffer (pH = 7), homogenized in a household blender for 2 minutes and strained through a nylon stocking. The final in vitro system containing 0.5ml of the slurry was added to 4.2 ml of the pre-reduced fermentation medium in 10 ml autoclaved fermentation bottles. Each batch culture consisted of 4.9 ml of the above slurry mixture to which 100 mg of IPE was added. At 0 and 24hrs, 800 μL of the aqueous volume was removed from each vial. For SCFA extraction, 100 μL of internal standard (IS; 2-ethylbutyrate, 73.8 mM) and 25 μL concentrated orthophosphoric acid was added to 225 μL of sample and thoroughly mixed and extracted with 3 x 1 mL of ether. The ether aliquots were pooled and a sub-sample transferred to a clean vial for analysis. Samples were analyzed by GC-FID (Trace GC, ThermoFisher, UK) using a ZB-WAX column (15 m × 0.53 mm × 0.25 μm; Phenomenex, Cheshire UK). The GC operating parameters were nitrogen carrier gas (1.89 ml/min) and GC oven parameters starting at an initial temp of 80 °C, ramp temp (10 °C /min) to 210 °C with splitless injection. The concentration of acetate, propionate and butyrate calculated using the area ratio to the IS and the calibrated response factor of each SCFA to the IS as determined by a gravimetrically prepared external standard.

*Esterase mediated propionate release from IPE*

An esterase basic kit containing esterases isolated from *Candida lipolytica*, *Mucor miehei*, *Pseudomonas fluorescens* (recombinant from *E. coli*), *Streptomyces diastochromogenes* (recombinant from *E.coli*), horse liver and hog liver was purchased from Sigma-Aldrich (Pool, UK). The lyophilized enzymes were defrosted and reconstituted in 100mM of phosphate buffer was added to each. The enzyme solutions were refrozen until required.

The enzyme solutions were defrosted prior to use. 100mg of IPE-27, 100mg ethyl propionate (EP) and 100mg inulin (negative control; lack of propionate release from inulin for each enzyme was verified in pilot work, data not shown) were each dissolved in 2ml phosphate buffer and incubated in a water bath (37°C) for 30 minutes. At T=0, 50uL of enzyme solution was added to the ester solution. Samples were run in triplicate.

In addition, 100mg inulin propionate ester and 2ml of buffer solution were added to two further vials and incubated as above. No enzyme was added to either vial. One vial was kept at room temperature to test how much propionate would be naturally released from the ester in the absence of the esterase. The second vial was heated to 80°C at the allotted sampling time-point for one hour in order to release all available propionate from the ester.

200uL of each reaction mixture was taken at T=0 (before the enzymes were added) and after 2 and 24 hours. 200uL of internal standard (IS; 10 mM butyric acid), 100uL orthophosphoric acid and 1ml diethyl ether were added to each sample. Immediately after the liquid phases separated, 400uL of the ether phase was extracted into a clean vial. 1uL of each sample was injected into a GC (HP5790, Palo Alto, USA) using a ZB-WAX column (30 m × 0.32 mm × 0.25 μm; Phenomenex, Cheshire UK) and He as carrier gas (1.8 ml/min) in split injection (~50:1 split ratio). The temperature program started at 40 °C and was held for 1 minute before ramping at 10 °C/min to 200 °C. Detection was carried out by flame ionization detection (FID). External standards were run daily to calculate the FID response factor and peak area ratios extracted to an Excel spreadsheet for data processing. Propionate concentration was calculated relative to the IS.

*Plasma PYY and GLP-1*

A cannula was inserted into an antecubital vein and baseline blood samples collected into heparin-coated tubes containing 0.2 mL of aprotinin (Sigma-Aldrich, Poole, UK) at −10 min and 0 min and then at 60 min intervals up 7 hours to assess plasma concentrations of PYY and GLP-1. Total GLP-1 and Total PYY were quantified using commercially available kits (GLP1T-36HK and PYYT-66HKRIA assay kits; Millipore, UK) following the manufacturers instruction for calibration and quantitation. PYY (pg/ml) and GLP-1 (pM) concentrations with time, area under the curve (AUC (pg.ml^-1^.hr) calculated by the trapedzoidal rule) and incremental AUC (iAUC, pg.ml^-1^.hr) were calculated. Results were compared using Kruskal-Wallis 1-way ANOVA in SPSS v22 (IBM Corp)

*Stool ^13^C analysis*

Volunteers were asked to collect the first post dose sample and return it to the investigation suite. Stools were homogenised and 2 aliquots (~3 g each) were stored at -20 °C until analysis. On the day of analysis, one aliquot was thawed and ~2 gram accurately weighted. Water (2 ml/g) was added and the vial thoroughly shaken to suspend the faecal sample. Immediately an aliquot (15 μL) was removed to a tin capsule (8 × 5 mm, Elemental Microanalysis, Oakhampton, UK) that had been prefilled with 20 μl of saturated sodium tetraborate and dried before addition of faecal material. The remaining faecal suspension was then centrifuged at 4000 × g for 20 minutes to remove cell material and undigested particulates. The faecal supernatant (50 μL) was then pipetted into tin boats containing dried borax. Boats were dried over a hotplate at 60 °C for one hour before crimping ready for elemental analysis-isotope ratio mass spectrometry. Briefly, the technique combusts the samples in a stream of O_2_, converting all carbon in the samples to CO_2_. The isotopic composition of the CO_2_ generated can then be measured by IRMS ^1^. Stool ^13^C output was expressed at percentage of the administered dose recovered per gram of stool wet weight (^13^C dose recovered % / g).

*Urine ^13^C analysis*

Collected urine samples were frozen at -20°C until analysis. For analysis, urine samples were diluted 1:20 with MilliQ water and transferred to 1.5ml crimp top vials. ^13^C/^12^C ratio in the urine (total C) was analysed by LC-IRMS as previously described ^2^ with minor modifications to the instrument setup. Briefly, the instrument was setup to exploit the auto-sampler and a column bypass set-up to allow direct inject to the IRMS oxidation interface. The samples were interspersed with an isotopically calibrated fucose standard which was used to back-calibrate the CO_2_ reference gas pulses that were introduced before and immediately after the sample CO_2_ peaks. From the measured ^13^C/^12^C ratio, results are reported calibrated against the international scale (VPDB) in units of δ^13^C (^0^/_00_).

#### *Palatability of IPE containing foods*

To test the acceptability of IPE, 15 subjects (5 males and 10 females) were recruited to assess the palatability of IPE-27 in two food matrices; chocolate drink and warm tomato soup. The chocolate drink comprised of 500g milk with 42 g of Nesquick, a chocolate flavor milkshake mix (Nestlé UK Ltd, Haxby Road, York, UK) containing no addition (control), 16.6 g inulin or 16.6g IPE-27. The soup was 194 g of canned tomato soup (Heinz, UK), warmed with nothing added (control), 10 g inulin or with 10 g IPE-27. Subjects consumed 20 ml of all milkshake and (separately) soup variants in a random order. Subjects were instructed to rinse their mouths with water in between tastings and were asked to complete a 100 mm visual analogue scale (VAS) to rate the pleasantness of each milkshake/soup (0mm = *Not at all pleasant*; 100 mm = *Extremely pleasant*).

*Appetite Visual Analogue Scales*

The VAS questionnaire was composed of 4 separate scales: 1) “*How hungry do you feel?”* 2) “*How full do you feel?”* 3) “*How strong is your desire to eat?”* 4) *“How sick do you feel?*” Each VAS consisted of a 100 mm line with opposing anchors “*Not at all”* and “*Extremely”* at 0 mm and 100 mm respectively. Subjects were asked to make a mark through each line to indicate their feelings at that given time.

**RESULTS**

**Inulin Propionate Ester**

*Chemical Characterization*

The characteristic vibration of an ester group was observed at 1736.8 cm^-1^ which was not present in the starting inulin product. GC analysis (n=12) yielded 1.25 ± 0.30 % free propionate of the total propionate yielded from the molecule on complete de-esterification. The ^13^C labelled IPE variants had a measured free propionate content of 2.20 and 11.63% for ^13^C IPE-27 and ^13^C IPE-54 respectively. Analysis of the total yield of propionate (and accounting for free propionate) from the molecule showed that the actual degree of esterification (d_e_) achieved was 0.74 ± 0.02.

*Purity*

Analysis of the salt content showed that overnight dialysis resulted in complete removal of the salt with some loss of ester yield but without compromising ester quality. In addition, the amount of free propionate was reduced by dialysis to <1% of the total propionate available after full de-esterification, resulting in a product that was almost tasteless when dissolved in water and undetectable when dissolved in fruit juice. Heavy metal screening showed that all IPE samples had heavy metal contents below that of the commercially sourced parent compound (< 1 mg/g for all species). The results of the microbiological testing indicated <20 cfu/g aerobic colony, <10 cfu/g enterobacteriaceae, <10 cfu/g E. Coli and “not detected” for Listeria (in 25 g) in all samples tested, well below the accepted thresholds for ready-to-eat foodstuffs ^2^.

*Esterase mediated propionate release from IPE*

Blank subtracted propionate release (as a percentage of the theoretical amount available from complete release) is shown in **Figure 1**. All of the esterase enzymes, except for *Mucor Miehei*, yielded net production of propionate over 24 hours although the yield from IPE was < 3% in all cases.

*Plasma PYY and GLP-1*

The results for plasma PYY (Figure 2A-C) and GLP-1 (Figure 2D-F) illustrate that fasting plasma PYY was significantly elevated for both IPE-27 (p = 0.035) and IPE-54 (p = 0.038) compared with inulin control. PYY iAUC was significantly lower for IPE-54 (p = 0.019) compared with inulin control but not for IPE-27 (p = 0.073, compared with inulin control). No significant differences were observed in fasting or postprandial GLP-1 responses.

*Stool ^13^C excretion*

Intake of IPE-54 and IPE-27 did not lead to a significant difference in ^13^C excretion in the stool samples (**Figure 3**, p > 0.05). The mean (SEM) stool output was 0.19 (0.15) and 0.55 (0.23) % for IPE-54 and IPE-27 respectively.

*Urine ^13^C excretion*

The ^13^C enrichment in urinary carbon is shown in **Figure 4**. There was no significant different in the abundance, shown as δ^13^C (^0^/_00_), between IPE-27 and IPE-54 treatments in 24hr urinary ^13^C output (p > 0.05).

*Palatability study*

The palatability of IPE in cold milk related products was indistinguishable from inulin and control but a significant reduction in palatability was observed when IPE was incorporated into heated products **(Figure 5)**.

*Appetite Visual Analogue Scales*

Intake of IPE in an identical evening meal and with a standardized breakfast the following morning did not significantly reduce hunger, satiety, fullness, hunger or desire to eat area under the curve (AUC) values compared to inulin control (p > 0.05, **Figure 6**). Repeated measures ANOVA did not reveal significant treatment × time effect in any of the subjective measures of appetite (**Figure 7**, p > 0.05).

**REFERENCES**

1. Preston T, McMillan DC. Rapid sample throughput for biomedical stable isotope tracer studies. Biomed Environ Mass Spectrom. 1988;16:229-35.
2. Morrison DJ, Taylor K, Preston T. Strong Anion Exchange Liquid Chromatography coupled with Isotope Ratio Mass Spectrometry using a Liquiface interface. Rapid Commun Mass Spectrom. 2010;24:1755-1762.
3. Gilbert RJ, Louvois J de, Donovan T, Little C, Nye K, Ribeiro CD, Richards J, Roberts D, Bolton FJ. Guidelines for the microbiological quality of some ready-to-eat foods sampled at the point of sale. Commun Dis Public Health 2000; 3:163-7.

**Figure Legends**

**Figure 1**. Propionate production from incubation of IPE-27 and ethyl propionate (EP; positive control) from esterases from *Candida lipolytica*, *Mucor miehei*, *Pseudomonas fluorescens* (recombinant from *E. coli*), *Streptomyces diastochromogenes* (recombinant from *E.coli*), horse liver and hog liver. Data are expressed and % of theoretical propionate recovery.

**Figure 2.** Plasma PYY concentration versus time (Figure 2A), plasma PYY AUC, AUC_0-180_, AUC_180-420_ and iAUC (Figure 2B) and plasma GLP-1 concentration versus time (Figure 2C), plasma GLP-1 AUC, AUC_0-180_, AUC_180-420_ and iAUC (Figure 2D). * indicates significant differences (p < 0.05) versus inulin control treatment.

**Figure 3.** Fractional stool ^13^C recovery x 100 (%) per gram wet weight. Participants followed a 6-day supplementation regimen followed with replacement of the dose on day 7 with either 100 mg of ^13^C-IPE-54 or 300 mg of ^13^C-IPE-27. In both studies, the carboxyl carbon (1-^13^C) of the propionyl moiety was labelled. Data are Mean ± SEM; n = 9.

**Figure 4**. 24hr urine ^13^C enrichment (abundance) after ingestion of ^13^C labelled IPE-27 and IPE-54 from the cross over trial. Data are expressed as mean (SEM) as δ^13^C (^0^/_00_).

**Figure 5.** Comparison of the organoleptic properties of control, inulin and inulin propionate (IPE-27) in chocolate milkshake (**A**) and heated tomato soup (**B**) (n = 15). Values are mean and SEM scores for visual analog scale assessment of how pleasant the material was to consume. ** p < 0.01 and * p < 0.05.

**Figure 6.** Subjective ratings of appetite following 7 day supplementation with inulin, IPE-54 and IPE-27. Ten grams of inulin ( ), IPE-54 ( ), or IPE-27 ( ) was ingested with an identical evening meal and with a standardized breakfast the following morning. **A.** *Hunger*, **B.** *Satiety*, **C.** *Fullness*, and **D.** *Prospective food consumption (PFC),* **E**. *Desire to eat.* Ratings were made using 100 mm visual analogue scales (VAS), with extreme statements anchored at each end of the rating scale (e.g. 0 mm *Not at all hungry*, 100 mm *Extremely hungry*). Data are mean ± SEM; n = 9.

**Figure 7.** Visual analogue scale area under the curve for the period 0 - 480 mins (AUC_0-480_; mm × min) data for each rating is also shown. Data are mean ± SEM; n = 9.

**Figure 1.**

**
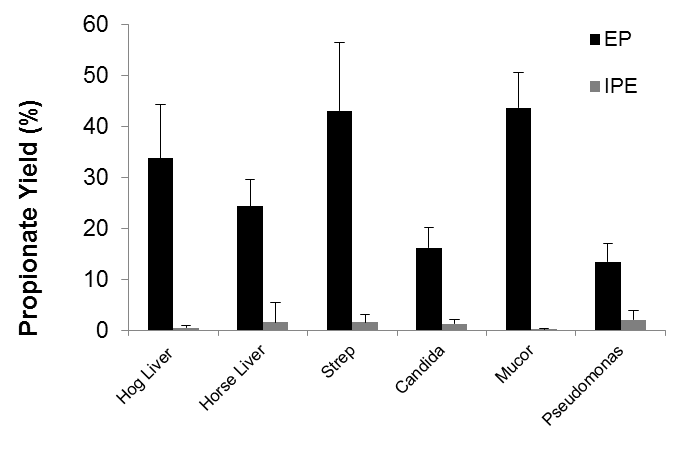
**

**Figure 2.**

**C**

**A**

*****

*****

*****

**D**

**B**

**Figure 3.**

**Figure 4.**

**Figure 5.**

**mm**

**mm**

**Figure 6.**

**Figure 7.**
